# Case Report: A case of focal segmental glomerulosclerosis in Wilson’s disease induced by penicillamine

**DOI:** 10.3389/fmed.2026.1802840

**Published:** 2026-03-30

**Authors:** Qi Li, Lili Yang, Xue Zheng, Yunjing Zhang

**Affiliations:** 1 Department of Nephrology, Zibo Central Hospital, Zibo, Shandong, China; 2 Department of Clinical Pharmacy, Zibo Central Hospital, Zibo, Shandong, China

**Keywords:** case report, drug-induced kidney injury, focal segmental glomerulosclerosis, nephrotic syndrome, penicillamine, podocytopathy, Wilson’s disease

## Abstract

Wilson’s disease (WD) is a hereditary disorder that impairs copper metabolism. Both WD and its treatment with penicillamine are associated with renal impairment. We report a case of penicillamine-induced podocytopathy with pathological findings of early-stage focal segmental glomerulosclerosis (FSGS), which demonstrated rapid reversibility upon drug withdrawal alone. A 36-year-old female of East Asian origin with a 12-year history of WD presented with nephrotic syndrome after 21 months of penicillamine therapy. Her 24-h proteinuria was 6.58 g. Her renal biopsy revealed a podocytopathy with early-stage FSGS lesions. Following discontinuation of penicillamine, the proteinuria decreased to 0.04 g/24 h and the nephrotic syndrome reached complete remission in 16 days without corticosteroid therapy. Remission persisted at the 1-year follow-up. This case illustrates that penicillamine can be a cause of reversible nephrotic syndrome secondary to podocytopathy with FSGS lesions and emphasizes the need for early biopsy in unusual proteinuria, with discontinuation of drugs as the main intervention. The clinician should have a high index of suspicion for drug toxicity in patients with WD and nephrotic syndrome, especially if the kidney injury is time-related to penicillamine therapy.

## Introduction

Wilson’s disease (WD) is a rare autosomal recessive disorder of copper metabolism (1). Renal involvement in WD is common and mostly presents with tubular dysfunction. Direct glomerular injury due to copper deposition occurs rarely (2, 3). In contrast, treatment of WD using penicillamine often causes glomerular disease, of which membranous nephropathy (MN) represents the most common pathological subtype (4, 5). Penicillamine-related focal segmental glomerulosclerosis (FSGS) within the spectrum of podocytopathy is less commonly reported. We report a case of penicillamine-induced podocytopathy with features of early-stage FSGS that rapidly resolved following the withdrawal of penicillamine alone, suggesting that this form of drug-induced injury can be reversible and emphasizing the importance of early recognition and drug discontinuation.

## Case presentation

A 36-year-old woman of East Asian origin with a 12-year history of WD was admitted to our hospital in April 2024 due to newly detected proteinuria (2+) over the preceding 3 days. In August 2022, the patient developed hepatic dysfunction and disease exacerbation with treatment using dimercaptosuccinic acid, bicyclol, and zinc sulfate. Accordingly, dimercaptosuccinic acid was discontinued and replaced with penicillamine (0.125 g/day), which was gradually increased to 0.5 g twice daily by October 2022. Proteinuria had been regularly monitored and remained non-significant until three days prior to admission, when she presented with 2+ proteinuria on her urinalysis. In August 2022, prior to initiating penicillamine therapy, her baseline eGFR was normal and urinalysis showed non-significant proteinuria (see Supplementary Table 1).

The patient presented on admission with normal blood pressure (109/72 mmHg), normal body shape, body mass index (BMI) of 22.1 kg/m^2^, and 3+ pitting edema in both lower limbs. She had no history of obesity or hypertension. She had no recent vaccinations or symptoms of infection. She denied use of any nonsteroidal anti-inflammatory drugs (NSAIDs), bisphosphonates, or other nephrotoxic medications during the relevant period. The laboratory findings on admission are summarized in Table 1. The patient’s 24-h urinary protein excretion was 6.58 g, serum albumin level was 19.4 g/L, and total serum cholesterol level was 7.9 mmol/L. Liver function and eGFR were normal. Serological tests for human immunodeficiency virus (HIV), hepatitis B virus (HBV), and hepatitis C virus (HCV) were negative. Her antinuclear antibody (ANA) test was positive at a titer of 1:100 (nuclear granular), and she tested positive for anti-SSA/Ro, anti-Ro-52, and anti-SSB/La antibodies. However, the absence of clinical manifestations such as xerostomia, xerophthalmia, or skin rash made primary Sjögren’s syndrome or systemic lupus erythematosus (SLE) unlikely.

**TABLE 1:** Results of laboratory examinations on admission.

Test	Result	Reference range
Blood tests		
Alanine aminotransferase, U/L	15.2	7.0–45.0
Aspartate aminotransferase, U/L	29.0	13.0–40.0
Gamma-glutamyl transferase, U/L	67.7	7.0–45.0
Albumin, g/L	19.4	40.0–55.0
Urea nitrogen, mmol/L	4.08	2.90–7.20
Creatinine, μmol/L	63.0	45.0–84.0
eGFR (CKD-EPI equation), mL/min/1.73 m^2^	111.56	90–120
Triglycerides, mmol/L	1.90	0.55–1.65
Total cholesterol, mmol/L	7.90	3.90–5.88
Copper, μmol/L	5.55	11.00–24.40
Ceruloplasmin, g/L	0.12	0.20–0.60
HIV antibody	Negative	Negative
HBV surface antigen	Negative	Negative
HCV antibody	Negative	Negative
ANA	Positive (1:100, nuclear granular)	Negative
Anti-dsDNA	Negative	Negative
Anti-SSA/Ro	2+ (36)	Negative
Anti-Ro52	3+ (65)	Negative
Anti-SSB/La	2+ (43)	Negative
Myeloperoxidase-ANCA	Negative	Negative
Proteinase-3-ANCA	Negative	Negative
Anti-GBM antibody	Negative	Negative
Anti-PLA2R antibody	<2.00	≤14
Urinary tests		
Protein	2+	Negative
Occult blood	1+	Negative
Red blood cell, /μL	6.0	0–17
24 h urine protein, g/24 h	6.583	0–0.150

eGFR, estimated glomerular filtration rate; CKD-EPI, Chronic Kidney Disease Epidemiology Collaboration; HIV, human immunodeficiency virus; HBV, hepatitis B virus; HCV, hepatitis C virus; ANA, antinuclear antibody; Anti-dsDNA, antibody to double-stranded DNA; ANCA, antineutrophil cytoplasmic antibody; GBM, glomerular basement membrane; PLA2R, phospholipase A2 receptor.

Nephrotic syndrome secondary to penicillamine therapy was suspected, and penicillamine was discontinued. With normal liver function, the hepatoprotective agent bicyclol was stopped. For long-term management of WD, the therapeutic regimen was transitioned to zinc sulfate monotherapy. Diuretics and human serum albumin were administered to address edema. On the sixth day of hospitalization, the 24-h urinary protein excretion increased to 11.28 g.

**FIGURE 1:**
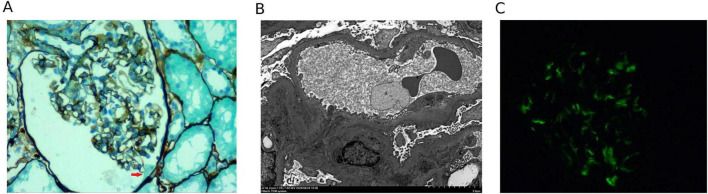
Pathological findings of the renal biopsy showing early-stage focal segmental glomerulosclerosis (FSGS) in the setting of podocytopathy. **(A)** Light microscopy showing adhesion of the glomerular tuft to the Bowman’s capsule (red arrow) in one glomerulus (PASM staining, ×400 magnification). **(B)** Electron microscopy showing diffuse effacement of podocyte foot processes with microvillous transformation of podocytes, no electron-dense deposits of diagnostic significance, and segmentally thickened GBM (TEM, ×2,500 magnification). **(C)** Immunofluorescence showing granular IgM deposits. PASM, periodic acid-schiff-methenamine; GBM, glomerular basement membrane; TEM, transmission electron microscopy.

A renal biopsy was performed on the sixth day. The results of the renal biopsy are presented in Figure 1. Nineteen glomeruli were identified in light microscopy with one showing an adhesion of the glomerular tuft to Bowman’s capsule, which is thought of as a nidus for segmental sclerosis (Figure 1A). The remaining glomeruli showed mild mesangial hyperplasia and podocyte vacuolization. Electron microscopy (Figure 1B) revealed diffuse foot process effacement and microvillous transformation of podocytes without electron-dense deposits. Immunofluorescence revealed IgM deposition (++) in a granular pattern (Figure 1C). These findings support a diagnosis of podocytopathy with features of early-stage FSGS. The absence of immune deposits supports a direct toxic effect on podocytes. IgM deposition is often considered non-specific in podocytopathies, likely representing trapped macromolecules in areas of injury rather than immune complex-mediated disease.

**FIGURE 2:**
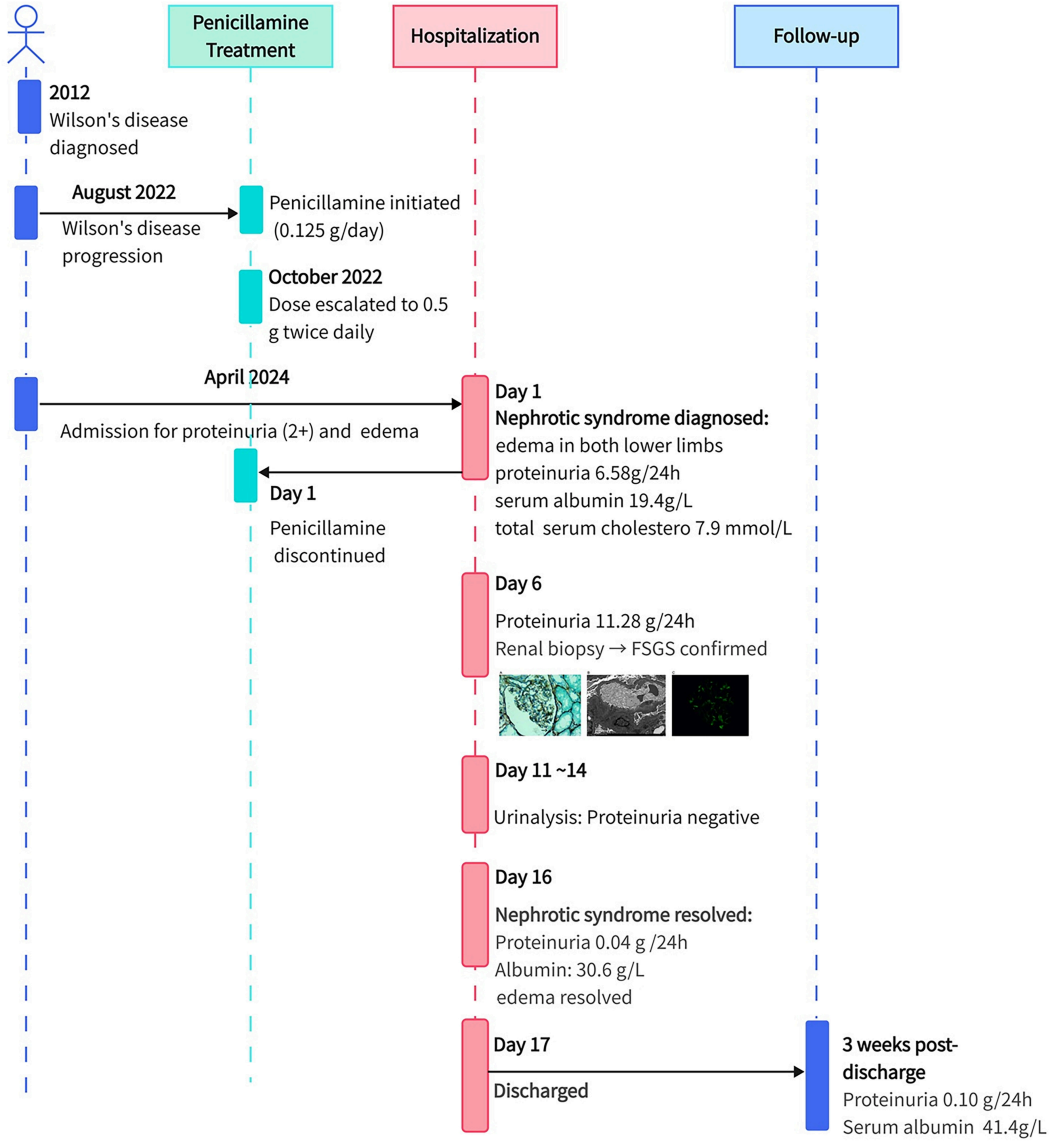
Timeline with the patient’s relevant data from this episode of care. The patient was diagnosed with Wilson’s disease in 2012. She began penicillamine therapy in August 2022. In April 2024, after 21 months of therapy, she was admitted with newly detected proteinuria (2+) and lower limb edema, and was diagnosed with nephrotic syndrome. Penicillamine was discontinued on hospital day 1. Proteinuria peaked at 11.28 g/24 h on day 6, when a renal biopsy confirmed podocytopathy manifesting as early-stage FSGS. The patient received no corticosteroids. Proteinuria became negative on day 14 and decreased to 0.04 g/24 h by day 16, with resolution of edema. Follow-up at 3 weeks showed sustained complete remission.

Potential corticosteroid therapy was discussed with the patient, but she declined, opting to observe whether her condition would resolve with the discontinuation of penicillamine alone. Notably, routine urinalysis on day 11 and day 14 showed normalized levels of proteinuria. On day 16, the 24-h urine protein level was 0.04 g, serum albumin level was 30.6 g/L, and total cholesterol level was 4.19 mmol/L. The edema in both lower limbs disappeared, and the patient was discharged on day 17. The serum creatinine level remained normal throughout the hospitalization period. The patient did not resume penicillamine or bicyclol after discharge. At 3-week follow-up, her 24-h urine protein level was 0.10 g and serum albumin level was 41.4 g/L, indicating significant improvement in nephrotic syndrome. She remained asymptomatic during subsequent follow-up. At the most recent 1-year telephone follow-up, she reported no recurrence of edema or other related symptoms, indicating sustained clinical remission. Timeline of patient’s historical and current information from this episode of care is shown in Figure 2. This case report was prepared in accordance with the CARE guidelines (6). The completed CARE checklist is provided in Supplementary Table 2.

## Discussion

We report a case of penicillamine-induced podocytopathy manifesting as early-stage FSGS in a patient with WD, which resolved completely and rapidly following penicillamine withdrawal. Renal complications in WD can be attributed to both the intrinsic nature of the disease and its treatment, particularly penicillamine (4). WD can directly lead to renal complications due to the direct toxicity of copper to renal tubular cells. Glomerular diseases directly caused by WD are rare, with only a few cases reported in literature (7–9). The most frequently described patterns are immunoglobulin (Ig) A nephropathy and IgM nephropathy, although a definitive causal relationship with WD remains to be established. FSGS has been reported even less frequently. Jin et al. (10) identified only one case of FSGS among seven patients with biopsy-proven renal damage attributed directly to WD. In that case, the patient’s massive proteinuria significantly improved after treatment with copper chelation therapy combined with corticosteroids. In contrast, the nephrotic syndrome in our patient rapidly resolved following the discontinuation of penicillamine treatment. This difference in therapeutic response makes it less likely that the FSGS in our patient was attributable to WD itself, and strongly supports a primary role of penicillamine toxicity.

Penicillamine, a penicillin derivative, functions as a copper-chelating agent and is the primary treatment for WD in many countries. However, its use is limited by significant adverse effects. In a retrospective study, 6.25% patients with penicillamine therapy developed proteinuria, making it the most common adverse event leading to permanent discontinuation of penicillamine (11). Nephrotic syndrome is a severe manifestation of renal impairment. Our patient developed nephrotic syndrome after 21 months of treatment with penicillamine which is consistent with findings in a previous study (5). Following the discontinuation of penicillamine, the patient’s nephrotic syndrome resolved completely in 16 days. This time to remission is shorter than the mean time of 7 months (range, 0.5–21) reported in the previous study (5). This discrepancy can be explained by the difference in the predominant pathological subtypes between the study cohort and our case. In that study (5), MN accounted for 55% of cases, whereas minimal change disease (MCD), a lesion on the same spectrum of podocytopathy as FSGS, accounted for 27%. The pathological findings in our patient were consistent with podocytopathy manifesting as FSGS, characterized by predominant podocyte injury in the absence of immune complex deposition. Both MCD and FSGS represent a spectrum of podocytopathy. The remarkably rapid resolution in our patient aligns with the expected behavior of this drug-induced podocytopathy spectrum (MCD/FSGS), in contrast to MN, where remission is often delayed due to the prolonged clearance of subepithelial immune complexes.

The causal relationship between FSGS and penicillamine in our patient was assessed using the Naranjo Adverse Drug Reaction Probability scale (12). The Naranjo Scale is a widely used tool in pharmacovigilance to determine the likelihood that a specific drug will cause an adverse drug reaction (ADR). The score for our patient was 7, indicating that FSGS was a probable adverse reaction to penicillamine (see Supplementary Table 3). Regarding the concomitant medication bicyclol, it is unlikely to have contributed to FSGS given the absence of reported glomerular toxicity. To further exclude its potential nephrotoxicity, we assessed the causal relationship between bicyclol and FSGS using the Naranjo scale (see Supplementary Table 3). Bicyclol received a score of 3, indicating a possible causal relationship with the renal injury. However, this score is limited by the concurrent withdrawal of penicillamine, which was the more definitive cause of the injury.

We performed a comprehensive search of PubMed, CNKI, and Wanfang Data (from inception to September 2025) using relevant Medical Subject Headings (MeSH) terms and keywords for penicillamine and FSGS/nephrotic syndrome. This search revealed only one published case report of FSGS associated with penicillamine (Table 2) (13). The use of corticosteroids in penicillamine-induced nephrotic syndrome varies in clinical practice (14). In the previous reported case (13), the patient who had rheumatoid arthritis and concurrent acute renal failure was treated with corticosteroids after the pathologic diagnosis of FSGS. In contrast, our patient with WD, who presented with nephrotic syndrome and normal renal function, achieved complete and rapid remission upon discontinuation of penicillamine without any immunosuppressive therapy. This indicated that penicillamine-induced podocytopathy presenting as FSGS without acute kidney injury can be reversible when penicillamine is discontinued early.

**TABLE 2:** Previously reported case of penicillamine-associated FSGS.

Feature	Details [Sugiyama et al. (13)]
Demographics	45-year-old female
Primary disease	RA
Penicillamine dose	100 mg/day
Time to symptom onset	Approximately 5 months after starting penicillamine
Clinical presentation	Nephrotic syndrome complicated by acute renal failure
Key laboratory findings at presentation	- 24-h urine protein: 37 g - Serum albumin: 1.5 g/dL - Serum creatinine: 2.7 mg/dL - Positive rheumatoid factor and ANA
Concomitant medications	NSAIDs, low-dose prednisolone (10 mg/day)
Renal biopsy diagnosis	FSGS
Intervention	1. Discontinuation of penicillamine. 2. Initiation of high-dose corticosteroid therapy (prednisolone 60 mg/day).
Outcome	Nephrotic syndrome and renal dysfunction recovered following the above intervention.

RA, rheumatoid arthritis; ANA, antinuclear antibody; NSAIDs, non-steroidal anti-inflammatory drugs; FSGS, focal segmental glomerulosclerosis.

The patient’s positive ANA, anti-SSA/Ro52, and anti-SSB antibodies raised concerns regarding underlying autoimmune disease. However, the absence of relevant clinical symptoms makes a primary autoimmune disease less likely. Importantly, penicillamine is known to induce autoantibody production as part of its immunomodulatory effects without causing clinically overt autoimmune diseases (15). The rapid and complete resolution of nephrotic syndrome following drug discontinuation, without corticosteroid therapy, strongly supports a direct drug-induced podocytopathy as the primary mechanism.

The pathogenesis of penicillamine-induced podocytopathy likely involves direct podocyte injury (16). Penicillamine could have direct toxicity upon podocytes through generation of free radicals. The thiol group of penicillamine chelates transition metals, such as copper, in podocytes, generating reactive oxygen species (ROS) that induce oxidative stress, lipid peroxidation and DNA damage, thus inducing podocyte apoptosis and dysfunction (17). Penicillamine has been shown to act as a hapten, inducing an autoimmune process involving dysregulated T-cell function and auto-antibody generation (18). Dysregulation of immunity could generate a milieu targeting proteins important for the podocyte cytoskeleton such as nephrin, causing podocyte damage and changes in glomerular permeability.

This was a single case report, which limits the generalizability of our conclusions to the general population. We cannot completely rule out the possibility of a very rare coincidental occurrence of idiopathic FSGS or WD-related FSGS. In addition, the follow-up period was relatively short for chronic glomerulopathy.

## Patient’s perspective

“I felt anxious when I faced the decision regarding the introduction of corticosteroids after being informed of the kidney biopsy results showing FSGS. The medical team explained carefully to me that my kidney injury had a strong temporal relationship with penicillamine and my renal function was still preserved, and discontinuation of penicillamine under close monitoring with no introduction of corticosteroids could work for me. I agreed with this plan after a thorough discussion. I felt relief that my proteinuria and edema disappeared within a few days. This experience has demonstrated that close attention needs to be paid to side effects of treatments and maintaining timely communication with the medical team is also important in chronic disease care.”

## Conclusion

Clinicians should maintain a high index of suspicion for drug toxicity in patients with WD and nephrotic syndrome, particularly when kidney injury is temporally associated with penicillamine exposure. This case expands the spectrum of penicillamine-induced kidney injury to include podocytopathy with FSGS lesions and underscores that such injury can be rapidly reversible upon drug discontinuation without immunosuppressive therapy. Therefore, in this context, early renal biopsy to confirm the diagnosis and prompt drug discontinuation are critical.

## Data Availability

The original contributions presented in this study are included in the article/Supplementary materials, further inquiries can be directed to the corresponding author.
