# Supplementary material for: Case Report: A case of focal segmental glomerulosclerosis in Wilson’s disease induced by penicillamine
Source: Front Med (Lausanne). 2026 Mar 30;13:1802840. doi: 10.3389/fmed.2026.1802840 (PMC13070819; doi:10.3389/fmed.2026.1802840)
Supplement: Supplementary file 1 [file Supplementary_file_1.docx]

Supplementary Material

# Supplementary Table 1 Baseline renal function and proteinuria prior to penicillamine therapy (August 2022)

| Test | Result | Reference Range |
| --- | --- | --- |
| Serum creatinine, μmol/L | 56.0 | 45.0–84.0 |
| eGFR (CKD-EPI equation), mL/min/1.73m² | 119.1 | 90-120 |
| Urinary Protein | negative | negative |

# Supplementary Table 2 CARE Checklist of information to include when writing a case report

| **Topic** | **Item No** | **Checklist item description** | **Reported on Section/Paragraph** |
| --- | --- | --- | --- |
| Title | 1 | The diagnosis or intervention of primary focus followed by the words “case report” | **Title**: "Case Report: A Case of Focal Segmental Glomerulosclerosis in Wilson's Disease Induced by Penicillamine" |
| Key Words | 2 | 2 to 5 key words that identify diagnoses or interventions in this case report, including "case report" | Before Abstract:” **Keywords:** case report, Drug-induced kidney injury, Focal segmental glomerulosclerosis, Nephrotic Syndrome, Penicillamine, Podocytopathy, Wilson's Disease” |
| Abstract  (Structured summary) | 3a | Background: state what is known and unknown; why the case report is unique and what it adds to existing literature. | **Abstract: “**Wilson’s disease (WD) is a …with renal impairment.” |
|  | 3b | Case Description: describe the patient’s demographic details, main symptoms, history, important clinical findings, the main diagnosis, interventions, outcomes and follow-ups. | **Abstract:“**We report a case of … at  the 1-year follow-up**.”** |
|  | 3c | Conclusions: summarize the main take-away lesson, clinical impact and potential implications. | **Abstract：**“This case illustrates… is time-related to penicillamine therapy.” |
| Introduction | 4 | One or two paragraphs summarizing why this case is unique **(may include references)** | **Introduction** |
| Patient Information | 5a | De-identified patient specific information | **Case presentation** section, first sentence: "A 36-year-old woman..." |
|  | 5b | Primary concerns and symptoms of the patient | **Case presentation** section: "...due to newly detected proteinuria (2+).." and subsequent description of edema and other symptoms. |
|  | 5c | Medical, family, and psycho-social history including relevant genetic information | **Case presentation** section: "A 36-year-old woman of East Asian origin with a 12-year history of WD..." and subsequent treatment history. |
|  | 5d | Relevant past interventions with outcomes | **Case presentation** section: "In August 2022, the patient... Accordingly ... by October 2022" |
| Clinical Findings | 6 | Describe significant physical examination (PE) and important clinical findings | **Case presentation** section, Paragraph 2: "The patient presented on admission... Her antinuclear antibody (ANA) test was positive ...." |
| Timeline | 7 | Historical and current information from this episode of care organized as a timeline | **Case presentation** section end: "Timeline of patient's... is shown in Figure 2." and the **Figure 2** legend. |
| Diagnostic Assessment | 8a | Diagnostic testing (such as PE, laboratory testing, imaging, surveys). | **Case presentation** section: Contains detailed description of laboratory results (Table 1) and renal biopsy findings (Figure 1). |
|  | 8b | Diagnostic challenges (such as access to testing, financial, or cultural) | Not explicitly detailed; differential diagnosis is discussed. **Discussion** section involves differentiation from FSGS caused by WD itself |
|  | 8c | Diagnosis (including other diagnoses considered) | **Case presentation** section: Describes the diagnosis of nephrotic syndrome and the renal biopsy-supported diagnosis of early-stage FSGS. Discussion “This difference in therapeutic response makes it less  likely that the FSGS in our patient was attributable to WD itself,  and strongly supports a primary role of penicillamine toxicity.”. |
|  | 8d | Prognosis (such as staging in oncology) where applicable | Not applicable |
| Therapeutic Intervention | 9a | Types of therapeutic intervention (such as pharmacologic, surgical, preventive, self-care) | **Case presentation** section: "Penicillamine was discontinued.", "...transitioned to zinc sulfate...", "Diuretics and human serum albumin..." |
|  | 9b | Administration of therapeutic intervention (such as dosage, strength, duration) | **Case presentation** section: Dosage and strength of diuretics and human serum albumin were not described, but the duration was described “the patient was discharged on day 17”. |
|  | 9c | Changes in therapeutic intervention (with rationale) | **Case presentation** section: Describes the rationale for discontinuing penicillamine and switching to zinc sulfate. “Nephrotic syndrome secondary to penicillamine therapy was suspected”. |
| Follow-up and Outcomes | 10a | Clinician and patient-assessed outcomes (if available) | **Case presentation** section: Details the course of proteinuria, serum albumin, edema, etc., after drug discontinuation and the final outcome. |
|  | 10b | Important follow-up diagnostic and other test results | **Case presentation** section: Describes urine protein and serum albumin results on days 11, 14, 16, and at 3-week and 1-year follow-ups. |
|  | 10c | Intervention adherence and tolerability (How was this assessed?) | Adherence implied by outcome; specific assessment method not detailed. The outcome (rapid resolution) indirectly reflects good adherence to drug discontinuation. |
|  | 10d | Adverse and unanticipated events | The case report focuses on the adverse event of penicillamine-induced FSGS. No new adverse events occurred after discontinuation. |
| Discussion | 11a | A scientific discussion of the strengths AND limitations associated with this case report | **Discussion** covers the uniqueness of the case and the inherent limitations of a single case report |
|  | 11b | Discussion of the relevant medical literature **with references** | **Discussion section:** Extensively references literature on WD nephropathy, penicillamine adverse effects, penicillamine induced FSGS etc. |
|  | 11c | The scientific rationale for any conclusions (including assessment of possible causes) | **Discussion section:** Uses the Naranjo scale (Supplementary Table 3) to assess causality. Discusses potential mechanisms (oxidative stress, immunology) from a pathophysiological perspective. |
|  | 11d | The primary “take-away” lessons of this case report (without references) in a one paragraph conclusion | **Conclusion section,** final paragraph. |
| Patient Perspective | 12 | The patient should share their perspective in one to two paragraphs on the treatment(s) they received | **Patient perspective section.** |
| Informed Consent | 13 | Did the patient give informed consent? Please provide if requested | **Yes ☑** No □ |

# Supplementary Table 3 Causal relationship between FSGS and penicillamine/bicyclol assessed by the Naranjo Scale

| Question | Yes | No | Do Not Know | Scores and reasons for penicillamine | Scores and reasons for bicyclol |
| --- | --- | --- | --- | --- | --- |
| 1.Are there previous conclusive reports on this reaction? | +1 | 0 | 0 | +1; There is one case report of FSGS possibly caused by penicillamine. Penicillamine induced nephrotic syndrome is a well-known adverse effect and has been reported in many literatures. | 0; No previous reports of bicyclol-induced FSGS or nephrotic syndrome in the literature or the label. |
| 2. Did the adverse event appear after the suspected drug was administered? | +2 | -1 | 0 | +2 The patient developed nephrotic syndrome two years after the initial of penicillamine administration. | +2; The patient developed nephrotic syndrome after the initial of bicyclol administration. |
| 3. Did the adverse event improve when the drug was discontinued or a specific antagonist was administered? | +1 | 0 | 0 | +1; The patient’s nephrotic syndrome resolved after discontinuing penicillamine for 16 days. | +1; The patient’s nephrotic syndrome resolved after discontinuing bicyclol along with penicillamine. |
| 4. Did the adverse event reappear when the drug was readministered? | +2 | -1 | 0 | 0; The patient didn’t receive a rechallenge of penicillamine. | 0; The patient didn’t receive a rechallenge of penicillamine. |
| 5. Are there alternative causes that could on their own have caused the reaction? | -1 | +2 | 0 | +2; Alternative causes of FSGS have been excluded. FSGS is not likely secondary to Wilson’s disease (see Discussion section). The patient doesn’t have obesity, HIV infection, heroin and other drugs that can cause secondary FSGS. | -1; Penicillamine is a known cause of podocytopathy (including FSGS). |
| 6. Did the reaction reappear when a placebo was given? | -1 | +1 | 0 | 0; Placebo challenge was not done. | 0; Placebo challenge was not done. |
| 7. Was the drug detected in blood or other fluids in concentrations known to be toxic? | +1 | 0 | 0 | 0; Drug levels were not available. | 0; Drug levels were not available. |
| 8. Was the reaction more severe when the dose was increased or less severe when the dose was decreased? | +1 | 0 | 0 | 0; The dose of penicillamine was not altered after the patient develop an adverse event. | 0; The dose of bicycol was not altered after the patient develop an adverse event. |
| 9. Did the patient have a similar reaction to the same or similar drugs in any previous exposure? | +1 | 0 | 0 | 0; The patient didn’t have any similar drugs. | 0; The patient didn’t have any similar drugs. |
| 10. Was the adverse event confirmed by any objective evidence? | +1 | 0 | 0 | +1; The patient’s manifestation and laboratory results was corresponding to nephrotic syndrome and renal biopsy pathology result was consistent with early-stage FSGS. | +1; The patient’s manifestation and laboratory results was corresponding to nephrotic syndrome and renal biopsy pathology result was consistent with early-stage FSGS. |
|  | **Total Score: 7** | | | | **Total Score: 3** |

The Naranjo scale comprises ten questions. Each question can be answered as “Yes”, “No”, or “Unknown”, with point values assigned to each answer. The final score interpretations are stratified into four categories: a score of ≥ 9 is considered “definite”, 5–8 “probable”, 1–4 “possible”, and ≤0 “doubtful” likelihood of the drug causing the ADR.
